# Usefulness of the Phase Angle in Evaluating Locomotive Syndrome in Cancer Patients

**DOI:** 10.3390/jcm14113980

**Published:** 2025-06-05

**Authors:** Ryoga Kashima, Ryo Yoshikawa, Wataru Saho, Yasumitsu Fujii, Risa Harada, Daisuke Makiura, Daisuke Tatebayashi, Katsuya Fujiwara, Mayu Mizuta, Junichiro Inoue, Yoshitada Sakai

**Affiliations:** 1 Division of Rehabilitation Medicine, Graduate School of Medicine, Kobe University, Kobe 650-0017, Hyogo, Japan; 2 Department of Physical Medicine and Rehabilitation, Kobe University Hospital, Kobe 650-0017, Hyogo, Japan; 3 Department of Rehabilitation Medicine, Kobe University Hospital, Kobe 650-0017, Hyogo, Japan

**Keywords:** cancer rehabilitation, phase angle, bioelectrical impedance, locomotive syndrome

## Abstract

**Background:** Locomotive syndrome (LS), a condition characterized by impaired mobility due to locomotive organ dysfunction, is highly prevalent among patients with cancer. The phase angle (PhA), measured via bioelectrical impedance analysis (BIA), reflects cellular health and nutritional status. This study aimed to investigate the association between LS and the PhA in patients with cancer. **Methods:** This cross-sectional study included hospitalized patients who underwent cancer treatment. The assessed variables included age, sex, body mass index (BMI), gait speed, grip strength, PhA, and the outcomes of LS risk assessment using the stand-up test, two-step test, and the 25-Geriatric Locomotive Function Scale (GLFS25). **Results:** A total of 190 patients (57 females, 133 males; mean age, 62.6 ± 17.2 years) were analyzed. The PhA was significantly negatively correlated with the LS stage (rs = −0.507, *p* < 0.001). Similarly, a significant negative correlation was observed between the PhA and each LS risk test, namely, the stand-up test, two-step test, and GLFS25. Furthermore, the PhA was identified as an independent and significant factor associated with LS progression (odds ratio, 0.361; 95% confidence interval, 0.221–0.588; *p* < 0.001). More effective and rapid than completing the full range of LS risk tests, measuring the PhA represents a convenient and practical tool for the early screening of mobility decline. **Conclusions:** The PhA is a simple and effective parameter for assessing mobility decline in patients with cancer. It is a potential clinical indicator for initiating rehabilitation interventions aimed at preventing the onset and progression of LS.

## 1. Introduction

Cancer continues to be a leading cause of mortality globally, with approximately 10 million cancer-related deaths reported annually [1,2]. However, advances in treatment approaches to cancer have significantly improved survival rates, resulting in an increasing number of patients who are living with cancer. Preserving the mobility of these individuals is thus essential, not only for the continuation of cancer therapy but also for preserving independence, even during end-of-life care [3]. Therefore, assessing mobility in patients with cancer is a critical component of comprehensive cancer care. The Eastern Cooperative Oncology Group (ECOG) Performance Status (PS) is a simple and widely used indicator that reflects the physical function of the patient, mobility, and overall condition [4]. However, PS does not always reflect the actual physical ability of the patient. Therefore, additional tools are needed that can more accurately assess mobility [5,6].

Locomotive syndrome (LS), a concept proposed by the Japanese Orthopedic Association (JOA) in 2007, refers to impaired mobility resulting from locomotive organ dysfunction [7]. In 2018, the JOA expanded its focus to include oncology, identifying LS in patients with cancer as a priority theme and emphasizing the importance of musculoskeletal management in cancer care. Previous studies have reported a high prevalence of LS among patients with cancer, with one study indicating that up to 96% of patients may be affected [8]. Therefore, preventing LS is as critical as maintaining PS.

Bioelectrical impedance analysis (BIA) is a widely used, noninvasive method for evaluating body composition. It operates by sending a weak electric current through the body to measure the resistance of different tissues, including fat, muscle, and bone. Among the various parameters derived from BIA, the phase angle (PhA) is considered one of the most clinically significant [9]. The PhA serves as a marker of cellular function [10] and has been associated with nutritional status, complications, prognosis, frailty, and sarcopenia in various diseases [11,12,13,14,15,16,17,18,19]. In addition, studies have shown that a lower PhA is associated with LS in general health-screening populations [20,21].

However, to the best of our knowledge, no study has investigated the relationship between the PhA and LS in patients with cancer. We hypothesized that the PhA is linked to the LS stage and is a contributing factor in its progression. This study aimed to evaluate the prevalence of LS in patients with cancer, examine the correlation between the PhA and LS stage, and identify factors associated with LS progression. If validated, our hypothesis supports the use of the PhA as a valuable parameter for assessing mobility decline in patients with cancer, and as a potential clinical indicator for initiating rehabilitation to prevent LS.

## 2. Materials and Methods

### 2.1. Study Design

This cross-sectional study was conducted at our institution between March 2022 and February 2025. Among the 2219 hospitalized patients with cancer who received rehabilitation during admission, 201 who provided informed consent were enrolled. All participants provided written informed consent. The study was conducted in accordance with the Declaration of Helsinki, and the protocol was approved by the Ethics Committee of the Kobe University Graduate School of Medicine (approval number B210225, 22 November 2021).

### 2.2. Measurement Items

Age, sex, primary tumor, height, and weight were recorded to calculate the body mass index (BMI). The BMI was categorized as underweight (<18.5 kg/m^2^), normal (18.5–25.0 kg/m^2^), or overweight (>25.0 kg/m^2^). Physical function was assessed based on the gait speed and grip strength. Gait speed was determined by timing a 4 m walk, whereas grip strength was measured twice for each hand in a seated position, with the highest value recorded. General conditions were assessed using the ECOG PS.

### 2.3. Assessment of Locomotive Syndrome (LS)

Before being assessed for LS, patients were required to undergo two physical performance tests—a stand-up test and a two-step test—and to complete a self-administered questionnaire, the 25-Geriatric Locomotive Function Scale (GLFS-25) [22]. In the stand-up test, the patients were asked to stand up from stools of varying heights (40, 30, 20, and 10 cm) using either one or both legs. The scoring system included nine performance levels: 0 (inability to stand); 1–4 (stand using both legs from a height of 40, 30, 20, and 10 cm); and 5–8 (stand using one leg from a height of 40, 30, 20, and 10 cm). Based on this score, the LS stages were assigned as follows: 5–8 (LS stage 0), 3–4 (stage 1), 2 (stage 2), and 0–1 (stage 3). In the two-step test, patients took two maximum-length steps, and the total distance was divided by the height to calculate a score. The LS stages were classified as ≥1.3 (stage 0), 1.1–1.3 (stage 1), 0.9–1.1 (stage 2), and <0.9 (stage 3). The GLFS25 assesses physical function and the living environment using 25 self-reported items, each scored from 0 to 4 points. The total scores were categorized as 0–6 (stage 0), 7–15 (stage 1), 16–23 (stage 2), and 24–100 (stage 3). Finally, patients were classified into LS stages ranging from 0 (no LS) to 3 (severe LS), based on the worst performance among the three tests. Patients with stage 1 or higher disease were diagnosed with LS.

### 2.4. Assessment of PhA

The PhA was measured using BIA with the InBody 770 device (InBody Co., Ltd., Seoul, Republic of Korea) [21]. This multifrequency device has been previously validated [23] and measures both whole-body and segmental PhAs. For this study, the whole-body PhA at 50 kHz was used. The patients stood barefoot on the device platform and gripped the hand electrodes to ensure full contact. Measurements were completed in approximately 1 min, and the PhA was automatically calculated using the following formula:

PhA (°) = (reactance/resistance) × (180°/π)

### 2.5. Statistical Analysis

All continuous variables are expressed as means ± standard deviations, and categorical variables are presented as counts and percentages. Spearman’s rank correlation coefficient was used to assess the correlation between the PhA and LS stage or LS risk test results. Ordinal logistic regression analysis was performed with the LS stage as the dependent variable and age, sex, BMI, grip strength, gait speed, and the PhA as the independent variables. The independent variables in the regression model were selected based on previous clinical studies indicating their potential influence on LS progression, rather than being determined solely by statistical significance in univariate analysis. All statistical analyses were performed using SPSS version 29.0 (IBM Corp., Armonk, NY, USA). In all analyses, a *p*-value of <0.05 was considered significant.

## 3. Results

### 3.1. Patient Characteristics

Among the 201 patients who provided informed consent, 190 patients with complete data were included in the final analysis. Table 1 summarizes the clinical characteristics of the patients. The mean patient age was 62.6 ± 17.2 years, with 30.0% of the participants being male. The most common primary cancers were hematological and gastrointestinal cancers. The mean BMI was 21.7 ± 3.5 kg/m^2^; mean gait speed, 1.15 ± 0.34 m/s; mean grip strength, 27.9 ± 8.7 kg; and mean PhA, 4.16 ± 0.78°.

### 3.2. Association Between LS and ECOG PS

**Figure 1:**
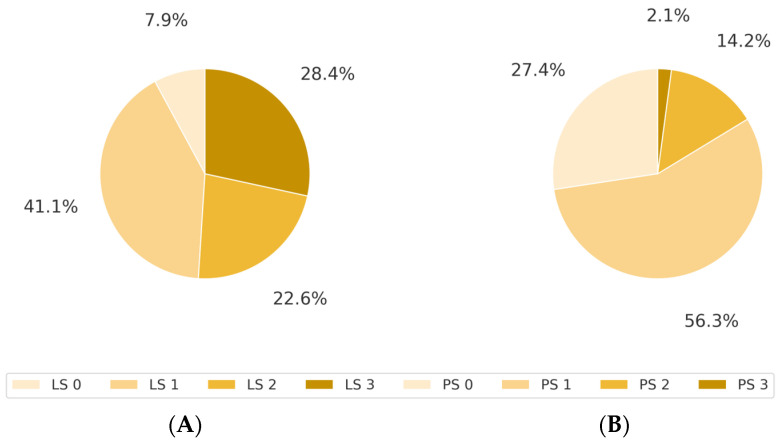
Pie charts showing the distribution of LS stages and ECOG PS. (**A**) Distribution of LS stages (0–3). (**B**) Distribution of ECOG PS (0–4).

The overall LS prevalence was 92.1%. LS stage 0 was observed in 7.9%, stage 1 was observed in 41.1%, stage 2 was observed in 22.6%, and stage 3 was observed in 28.4% of the patients (Figure 1A). The distribution of ECOG PS was as follows: PS 0, 27.4%; PS 1, 56.3%; PS 2, 14.2%; and PS 3, 2.1%; no patient was classified as PS 4 (Figure 1B). Among patients with PS 0, 76.9% (40/52) had LS. This proportion increased to 97.2% (104/107) for PS 1 and 100% for PS 2 and 3 (Table 2).

### 3.3. Distribution of PhA According to LS Stage

**Figure 2:**
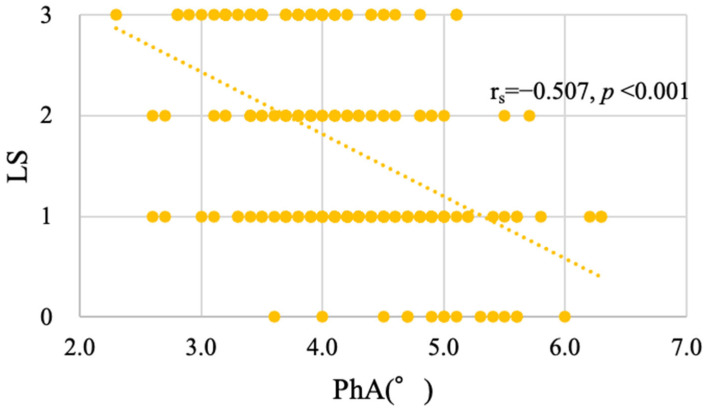
Scatter plot illustrating the relationship between the PhA and LS stages. *X*-axis, PhA (°); *Y*-axis, LS stages.

**Figure 3:**
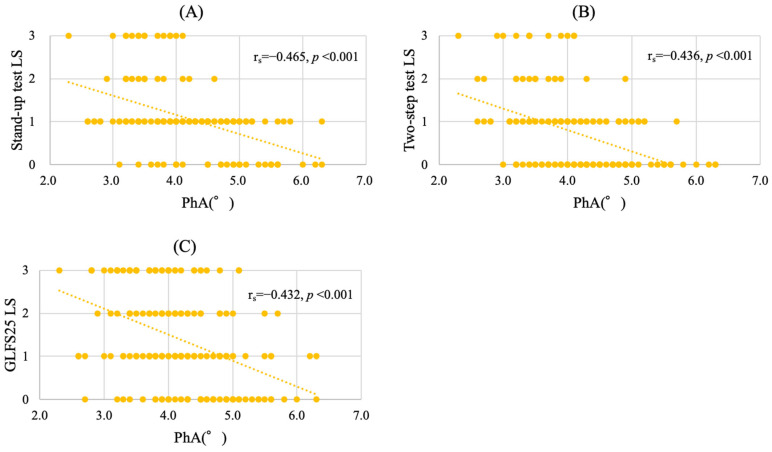
Scatter plots illustrating the relationship between the PhA and LS stages in three tests: stand-up test (**A**), two-step test (**B**), and GLFS25 (**C**). *X*-axis, PhA (°); *Y*-axis, LS stages.

The PhA was significantly and negatively correlated with the LS stage (r_s_ = −0.507, *p* < 0.001) (Figure 2). As the LS stage progressed, the mean PhA values decreased to 4.99°, 4.41°, 4.01°, and 3.68° at stages 0, 1, 2, and 3, respectively. The PhA was also negatively correlated with the stand-up test (r_s_ = −0.465, *p* < 0.001) (Figure 3A), the two-step test (r_s_ = −0.436, *p* < 0.001) (Figure 3B), and the GLFS25 scores (r_s_ = −0.432, *p* < 0.001) (Figure 3C).

### 3.4. PhA as an Associated Factor of LS Progression in Cancer Patients

The PhA was an independent and significant factor associated with LS progression (odds ratio [OR]: 0.361; 95% confidence interval [CI]: 0.221–0.588; *p* < 0.001). Additionally, gait speed (OR: 0.235, 95% CI: 0.086–0.642, *p* = 0.005) and grip strength (OR: 0.931, 95% CI: 0.880–0.984, *p* = 0.012) were independently and significantly associated with LS progression (Table 3). Multicollinearity was assessed using the variance inflation factor, and no significant issues were observed.

## 4. Discussion

In this study, the PhA was identified as an independent and significant factor associated with the progression of LS in patients with cancer. The PhA showed a significant negative correlation with the LS stage and with each of the three LS risk tests, and the mean PhA progressively declined with the advancement of LS.

The prevalence of LS among cancer patients is extremely high [8]. A previous study that used propensity score matching found an approximately 89% prevalence of LS in cancer patients [24]. In alignment with these findings, the present study observed a high LS prevalence of 92.1%, indicating a substantial decline in mobility among patients with cancer.

LS in cancer patients can be classified into three distinct types [4]. The first type is directly caused by the cancer itself, the second by the cancer treatment, and the third by coexisting locomotive dysfunction. LS in cancer patients is often multifactorial, with multiple types potentially coexisting [8]. Therefore, LS in patients with cancer is more complex than in the general population, tends to progress more rapidly, and is more likely to reach severe stages. These findings highlight the urgent need for early screening and intervention to mitigate mobility decline in cancer patients.

Regarding the relationship between ECOG PS and LS, 76.9% of patients classified as having PS 0 were found to have LS. This proportion increased to 97.2% for PS 1 and reached 100% for PS 2 and 3. These results indicate that LS risk tests may be more sensitive in detecting early-stage mobility decline than ECOG PS alone. Moreover, even when PS appears intact, patients may experience substantial reductions in mobility, emphasizing the importance of LS risk tests in this population.

Previous studies have reported the reference values for the PhA in healthy individuals. For example, among healthy Asian individuals aged 18–94 years, the average PhA was 6.55° [25], and in another study involving healthy individuals aged 18–50 years, the mean PhA was 7.321° [26]. In contrast, the mean PhA in the cancer patient cohort in this study was 4.16°, suggesting that the PhA in these patients was significantly lower as compared to the healthy population.

The clinical utility of the PhA in patients with cancer has been previously reported [27,28,29,30,31], and studies in general health-screening populations have shown an association between the PhA and LS [20,21]. However, to the best of our knowledge, no previous studies have investigated the relationship between the PhA and LS in patients with cancer. This is the first study to demonstrate that the PhA is significantly correlated with LS and serves as an independent factor associated with LS progression in patients with cancer. As measuring the PhA is simpler than completing the full range of LS risk assessments, it could serve as a convenient and practical tool for evaluating mobility decline in patients with cancer. Furthermore, when the PhA is low, the early initiation of rehabilitation may be necessary, even in patients whose PS has not yet declined.

This study had certain limitations. First, this was a single-center cross-sectional study, and clinical outcomes could not be followed. Future studies including longitudinal clinical data from larger patient populations are warranted. Second, the study sample was limited to inpatients undergoing both cancer treatment and rehabilitation, which introduced a potential selection bias. Outpatients and patients who did not undergo rehabilitation were excluded. Third, the BIA measurements were conducted without standardizing physiological conditions, such as fasting status, hydration level, and bladder fullness. These factors may have affected the phase angle values and introduced variability into the results. Finally, a wide variety of cancer types were identified in this study. Therefore, further studies that focus on specific primary cancer sites are required.

## 5. Conclusions

The prevalence of LS among cancer patients is extremely high, indicating that a significant proportion of patients experience a substantial decline in mobility. For the first time, we demonstrated that the PhA progressively decreases with advancing LS stages and is significantly associated with LS progression. As the PhA is easily measurable and reflects mobility impairment, it may serve as a useful clinical parameter for assessing LS risk and determining the optimal timing for initiating rehabilitation aimed at preventing the onset and progression of LS in patients with cancer.

## Figures and Tables

**Table 1:** Patient characteristics.

	Total (n = 190)	Percentage
Age (years)	62.6 ± 17.2	
Sex		
Female	57	30.0
Male	133	70.0
Primary tumor		
Leukemia	54	28.4
Lymphoma	31	16.3
Esophagus	29	15.3
Gastric	28	14.7
Multiple myeloma	10	5.3
Colorectal	6	3.2
Others	32	16.8
BMI (kg/m^2^)	21.7 ± 3.5	
Underweight < 18.5	33	17.4
Standard 18.5–25.0	123	64.7
Obesity > 25.0	34	17.9
Gait speed (m/s)	1.15 ± 0.34	
Grip strength (kg)	27.9 ± 8.7	
Phase angle (°)	4.16 ± 0.78	

**Table 2:** Correspondence between LS stage and ECOG PS.

	LS 0	LS 1	LS 2	LS 3	Total
PS 0	12	28	6	6	52
PS 1	3	44	33	27	107
PS 2	0	6	4	17	27
PS 3	0	0	0	4	4
PS 4	0	0	0	0	0
Total	15	78	43	54	190

**Table 3:** Ordinal logistic regression analysis identifying factors associated with LS progression.

Variable	OR	95% CI	*p*-Value
Phase angle	0.361	0.221–0.588	<0.001
Gait speed	0.235	0.086–0.642	0.005
Grip strength	0.931	0.880–0.984	0.012
Age	1.003	0.986–1.021	0.704
Sex			
Female	0.635	0.285–1.413	0.266
Male	1.000	-	-
BMI			
Underweight	1.358	0.447–4.124	0.59
Standard	0.946	0.430–2.081	0.89
Obesity	1.000	-	-

OR: odds ratio, CI: confidence interval.

## Data Availability

The data supporting the findings of this study are available upon request from the corresponding author.

## Abbreviations

LS	locomotive syndrome
BIA	bioelectrical impedance analysis
PhA	phase angle
BMI	body mass index
GLFS25	25-Geriatric Locomotive Function Scale
ECOG	Eastern Cooperative Oncology Group
JOA	Japanese Orthopedic Association
PS	performance status
